# The therapeutic potential of targeting the endothelial-to-mesenchymal transition

**DOI:** 10.1007/s10456-018-9639-0

**Published:** 2018-08-03

**Authors:** Shirley Man, Gonzalo Sanchez Duffhues, Peter ten Dijke, David Baker

**Affiliations:** Department of Cell and Chemical Biology and Oncode Institute, Leiden University Medical Center, Einthovenweg 20, 2300 RC Leiden, The Netherlands

**Keywords:** Endothelial cell, Endothelial-to-mesenchymal transition, Differentiation, Signaling, Tissue engineering, TGF-β, Vascular disease

## Abstract

Endothelial cells (ECs) have been found to be capable of acquiring a mesenchymal phenotype through a process known as endothelial-to-mesenchymal transition (EndMT). First seen in the developing embryo, EndMT can be triggered postnatally under certain pathological conditions. During this process, ECs dedifferentiate into mesenchymal stem-like cells (MSCs) and subsequently give rise to cell types belonging to the mesoderm lineage. As EndMT contributes to a multitude of diseases, pharmacological modulation of the signaling pathways underlying EndMT may prove to be effective as a therapeutic treatment. Additionally, EndMT in ECs could also be exploited to acquire multipotent MSCs, which can be readily re-differentiated into various distinct cell types. In this review, we will consider current models of EndMT, how manipulation of this process might improve treatment of clinically important pathologies and how it could be harnessed to advance regenerative medicine and tissue engineering.

## Introduction

A substantial body of experimental evidence has shown that epithelial cells possess the intrinsic capability to become mesenchymal cells in a process called epithelial-to-mesenchymal transition (EMT) [1, 2]. EMT is a reversible cell differentiation event associated with extensive alterations at the transcriptional, translational, and morphological level. It is an essential physiological mechanism which is indispensable for several stages of embryogenesis [3] as well as wound healing [4], but it can also promote pathological phenomena such as cancer metastasis and fibrosis [1]. In the past few years, endothelial cells (ECs) have also been found to undergo a similar dedifferentiation process known as endothelial-to-mesenchymal transition (EndMT) [5]. Throughout this highly dynamic process, ECs progressively dedifferentiate into mesenchymal stem-like cells (MSCs) and acquire the characteristics of multipotent cells. During EndMT, ECs spawn a wide spectrum of intermediate phenotypes [6]. These changes in differentiation status  and cell behavior are illustrative of their inherent plasticity since their ability to transition is reversible (i.e., mesenchymal-to-endothelial transition) and the process can be either full or partial [7].

ECs that undergo EndMT are characterized by a phenotypic switch involving: (i) loss of cellular adhesion due to the downregulation of proteins involved in cell–cell junctions; (ii) cytoskeletal reorganization, which converts tightly compacted cobblestone-like cells into spindle-shaped cells with no apical-basal polarity [5]; (iii) reduced expression of distinctive EC markers, such as vascular endothelial (VE)-cadherin, CD31/PECAM-1, TIE1, TIE2, and von Willebrand Factor (vWF); (iv) increased expression of mesenchymal cell markers, such as fibroblast-specific protein-1 (FSP-1), alpha-smooth muscle actin (α-SMA), vimentin, and N-cadherin [8]. EndMT-derived cells thus exhibit an enhanced migratory potential and increased extracellular matrix (ECM) production, both of which are hallmarks of invasive cells [9, 10].

EndMT was first observed in the developing embryo, where it was shown to occur in subsets of ECs during cardiogenesis and vasculogenesis. ECs in the endocardium undergo EndMT, invade the cardiac jelly and eventually generate the cardiac cushions. Disruption of EndMT at this embryonic stage results in abnormal formation of the cardiac valves and embryonic lethality [11–13]. Similarly to EMT, EndMT can be triggered postnatally under certain pathological conditions, such as tissue damage or inflammation, thereby giving rise to fibroblasts and myofibroblasts [14]. Through the combination of genetic labeling of ECs and disease animal models [5], EndMT was demonstrated to contribute to wound healing [12], pulmonary arterial hypertension (PAH) [15], atherosclerosis [16], cardiac and renal fibrosis [12, 17, 18], fibrodysplasia ossificans progressiva (FOP) [19], and cancer progression [10]. Accordingly, most EndMT research has focused on its role in disease and approaches to block this process. By example, recently, researchers have used small molecules to modify the signaling pathways governing EndMT in an attempt to inhibit or reverse its effects [5]. Interestingly, EndMT could also be used in a different manner, wherein ECs may be exploited to derive multipotent MSCs, which can be readily re-differentiated into various distinct cell types [20].

Here, we will review the evidence that EndMT is integral to the development and evolution of certain pathologies and that targeting EndMT represents a potential therapeutic avenue to treat disease. First, we will describe the signaling pathways that stimulate ECs to undergo EndMT, including the inhibitory mechanisms that prevent this mesenchymal transition. Next, we will discuss how EndMT has been targeted in different disease contexts. Finally, the potential for exploiting EndMT in regenerative medicine and tissue engineering will be assessed.

### EndMT-promoting mechanisms

The extent to which ECs lose their distinctive characteristics and gain mesenchymal properties is dependent on the tissue and signaling contexts. It is established that numerous different stimuli can promote EndMT. Below, some of the principal pro-EndMT cues are considered.

#### Signaling pathways

As ECs share a number of characteristics with epithelial cells (e.g. apical-basal polarity, tight cell junctions, absence of migratory features), it  is reasonable to assume that EndMT is related to the process of EMT, and is thus modulated by many of the same pathways and effectors [7]. Ultimately, activation of these pathways results in the expression of common transcription factors, such as Snail, Slug, Twist, ZEB1, ZEB2, and Sox2 [5, 17, 20, 21]. These well-characterized transcription factors initiate EndMT, likely by repressing the expression of endothelial genes (e.g. *CDH5* and *PECAM1*) and subsequently activating the expression of mesenchymal genes (e.g. *VIM* and *COL5A1*) [22], thereby transforming ECs into a mesenchymal state.

The best-studied mediators of EndMT are the transforming growth factor (TGF)-β and bone morphogenetic protein (BMP) family of growth factors, which signal through both Smad-dependent and Smad-independent pathways [23, 24]. This diverse superfamily of proteins (i.e. TGF-βs, BMPs, activins, and growth differentiation factors (GDFs)) exert pleiotropic effects in most, if not all, tissues and are indispensable for many physiological processes, including inflammation and wound repair [25]. Members of the TGF-β family signal via specific receptor complexes at the cell membrane. An archetypal response is illustrated by TGF-β1, which binds with high affinity to the type II TGF-β receptor (TGF-βRII) resulting in the recruitment and phosphorylation-dependent activation of the type I TGF-β receptor (activin receptor-like kinase (ALK) 5) [5]. The active ALK5 binds and phosphorylates Smad2/3, which interacts with Smad4 to form a transcription complex that translocates to the nucleus and triggers the expression of specific genes [6, 23]. This subset of genes includes those upregulated in EndMT, such as *NOTCH1, TWIST1*, and *SNAI1*/*2* [22]. In addition, certain TGF-β family members (TGF-β2, BMP2, and BMP4) were found to induce EndMT by signaling through ALK2 [23, 26]. In vivo relevance of this mechanism is illustrated by the EC-derived heterotopic ossification observed in patients with FOP, which is due to an overactive mutant ALK2 [6, 26]. The pivotal role that the TGF-β superfamily plays in the initiation of EndMT has not only been observed in vitro [5, 6, 27, 28], but has also been validated in multiple in vivo mice studies, which showed that the knockdown and knockout of several TGF-β signaling-related genes, such as *SMAD2, SMAD3*, and *TGFBR2*, prevented EndMT [29, 30].

TGF-β signaling can induce EndMT either directly, as described above, or indirectly, as exemplified by the Wnt pathway, caveolin-1 (CAV1), and endothelin-1 (ET-1). The Wnt pathway comprises a multigene family of secreted glycoproteins that play important roles during embryogenesis and heart cushion development [31, 32]. Several studies have confirmed the involvement of Wnt proteins in the induction of EndMT via Smad-dependent TGF-β signaling, and canonical (i.e. involving β-catenin) and non-canonical Wnt signaling pathways [33–36]. Additionally, studies have found that canonical Notch signaling can act in concert with TGF-β to induce EndMT by activating expression of Snail [37–39]. It should be noted that Kaposi’s sarcoma-associated herpesvirus was found to cause EndMT via Notch signaling independently of the TGF-β pathway [40]. Caveolin 1 (CAV1) is the major component of caveolae that controls TGF-β signaling by internalizing, trafficking, and degrading TGF-β receptors [41]. Mice lacking CAV1 undergo spontaneous EndMT, which can be augmented by treatment with TGF-β [42]. Finally, recent studies using human ECs have demonstrated that ET-1, an endogenous vasoconstrictor polypeptide, can stimulate EndMT and yield myofibroblasts, either alone or in combination with TGF-β [43–45].

#### Inflammation, metabolic status, and shear stress

Several lines of evidence support the view that inflammation, metabolic status, and shear stress can all strongly influence EndMT. Firstly, proinflammatory molecules such as interleukin (IL)-1β, IL-6‚ interferon (IFN)-γ, and tumor necrosis factor (TNF)-α have been shown to stimulate EndMT by activating expression of Snail and Slug in synergy with TGF-β [46–48]. Secondly, matrix metalloproteinases (MMPs) play a role in several physiological processes and contribute to tissue homeostasis and remodeling, and also function during inflammation by regulating various cytokines, chemokines, and ECM proteins [49]. They are known to initiate EMT through cleavage of cell–cell junction proteins, and, more recently, have been shown to be associated with EndMT [22, 50, 51]. Thirdly, recent studies demonstrated that EndMT was induced by hypoxia via activation of Snail, and hypoxia-inducible factor-1 α (HIF-1α) was observed to promote the process during the development of radiation-induced pulmonary fibrosis [52, 53]. Additionally, HIF-1 has been shown to increase the levels of platelet-derived growth factor (PDGF)-β) and TGF-β1 signaling leading to EndMT via downregulation of neprilysin (NEP) [54]. Differential oxygen concentrations drive EndMT in a different manner. Reactive oxygen species (ROS) are a byproduct of oxygen metabolism whose levels fluctuate as a consequence of environmental stresses (e.g temperature changes and UV light). ROS stimulate EndMT, e.g. by inducing TGF-β expression, which in turn leads to the production of ROS via a positive feedback loop [55]. ROS also activate nuclear factor-κΒ (NF-κB) signaling, which drives EndMT in synergy with TGF-β [56]. Furthermore, NADPH oxidase 4 (NOX4), an enzyme responsible for the production of ROS, was found to mediate TGF-β-dependent production of myofibroblast by EndMT [57, 58]. Recently, the Akt/mammalian target of rapamycin (mTOR)/70 kDa ribosomal S6 kinase (p70S6K) signaling pathway was also shown to be involved in TGF-β1-induced EndMT in transplant kidney interstitial fibrosis [59]. Finally, hemodynamic forces have been demonstrated to strongly modulate EndMT [60]. Shear stress, a fundamental force governing homeostasis of ECs, suppresses EndMT via a number of TGF-β signaling-dependent mechanisms [61]. Correspondingly, whereas high shear stress appears to inhibit EndMT [61], disturbed flow is a potent EndMT inducer *in vivo* as well as in organ-on-a-chip devices. Under these conditions, genetic inhibition of extracellular-signal-regulated kinase (ERK) 5 signaling enhances EndMT, whereas ERK5 overactivation prevents EndMT in cells exposed to disturbed flow or stimulated by TGF-β in static conditions [62]. A different mechanical stress, termed cyclic strain, and caused by a perpendicular stretching force on the vessel wall, has been shown to potentiate EndMT by augmenting both TGF-β and Wnt signaling [63, 64].

#### microRNAs

MicroRNAs (miRNAs) control EndMT by altering the activity of signaling intermediates leading to changes in signaling amplitude and output. miRNA 125b has been shown to contribute to EndMT progression [65], and it has been demonstrated that miRNA21 mediates TGF-β-induced EndMT by controlling actin remodeling and promoting the secretion of inflammatory cytokines [66]. Several other miRNAs were also found to be positive modulators of EndMT, such as miR-31, which is required for the expression of EndMT markers following TGF-β-treatment [67], and miR-9, a miRNA regulated by TNF-α signaling [68]. Additionally, metastasis-associated lung adenocarcinoma transcript 1 (MALAT1), a long non-coding RNA, was found to modulate TGF-β1-induced EndMT of endothelial progenitor cells (EPCs) through regulation of TGF-βRII and Smad3 via decreased miR-145 expression [69].

### EndMT-inhibiting signaling pathways and mechanisms

In addition to stimuli favoring EndMT, there are also a number of different factors involved in the negative regulation of this process.

#### Signaling pathways

Although TGF-β and BMP are known to induce EndMT under specific conditions, they can also bind ALK1 to activate Smad1/5/8, which induces proliferation at the expense of EndMT [70]. Endoglin, an accessory type III TGF-β receptor, partially regulates the equilibrium between ALK1/ALK5 activation. By stimulating downstream Smad1/5/8 responses it can indirectly inhibit ALK5 signaling, and thus inhibit EndMT [71]. Interestingly, BMP7 appears to be a negative regulator of EndMT [72], presumably through the activation of ALK2 alone (and the associated Smad1/5/8 pathway), in contrast to BMP2 and BMP4 which bind to ALK2 in conjunction with ALK5 and thereby promote EndMT [6, 26].

Another known mechanism of EndMT inhibition is vascular endothelial growth factor A (VEGF-A)-stimulated VEGF receptor (VEGFR)2 signaling [73]. This process, however, is counteracted by VEGF-A sequestration by VEGFR1, thereby preventing its interaction with VEGFR2, and leading to EndMT [74]. Two other layers of regulation of this network are repression of VEGF-A by BMP signaling [75], and attenuation of VEGF-A signaling by mechanical cyclic strain [19].

Other signaling cascades and factors that abrogate EndMT include: (i) activation of the Src signaling pathway by hydrogen sulfide during endoplasmic reticulum (ER) stress [76]; (ii) glucagon-like peptide-1 (GLP-1) suppression of hyperglycemia-induced EndMT via reduced expression of ROS and inhibition of ROS-activated poly(ADP-ribose) polymerase 1 (PARP-1) [77]; (iii) high-density lipoprotein (HDL) inhibition of TGF-β1-induced EndMT [78]; (iv) endothelial heat shock protein beta-1 (HSPB-1)-mediated EndMT inhibition after stimulation with fibrotic cytokines [79]; (v) netrin-1-mediated attenuation of EndMT during renal dysfunction, as demonstrated in a nephrectomy rat model [80]; (vi) expression of ECM protein fibulin-1 via reduced expression of TGF-β2 [81]; (vii) secretion of cytokines and angiogenic factors by macrophages sustains endothelial differentiation of EPCs and consequently restricts EndMT during muscle regeneration [82].

#### miRNAs

miRNAs have been shown to block EndMT in numerous different tissues. Several miRNAs, such as miR-15a, miR-23b, and miR-199a, have been found to impair EndMT during heart development [83]. TGF-β-induced EndMT was blocked by miR-126 in bone marrow-derived EPCs through direct targeting of the phosphoinositide 3-kinase (PI3K) subunit p85 [84]. miR-155 was found to be a potent inhibitor of TGF-β-induced EndMT via inhibition of RhoA expression [85, 86]. Furthermore, miR-302c was observed to suppress EndMT in hepatocellular carcinoma by negatively regulating the expression of metadherin (MTDH) [87]. Fibroblast growth factor receptor 1 (FGFR1) signaling can also inhibit TGF-β-induced EndMT by promoting the expression of miRNA let-7, a negative regulator of TGF-β signaling [88]. *N*-acetyl-seryl-aspartyl-lysyl-proline (AcSDKP), a peptide substrate of angiotensin-converting enzyme (ACE), contributes to this by upregulating let-7 and restoring FGFR levels [89]. FGF-2, although found to induce EndMT in some types of ECs [90], has also been demonstrated to abrogate TGF-β-induced EndMT through miR-20 [91]. Lastly, miR-630 was shown to inhibit EndMT in heterotopic ossification by targeting Slug [92].

#### Autophagy

Recently, autophagy has emerged as a potentially important player in controlling EndMT by decreasing TGF-β2-induced EndMT [93]. Activation of autophagy was also shown to reduce expression of Snail by decreasing the phosphorylation levels of Smad3, thus counteracting EndMT [94]. Furthermore, pharmacological inhibition of mTOR resulted in the activation of autophagy and a decrease of EndMT [95], providing evidence of a causal link between mTOR-dependent inhibition of autophagy and EndMT. These findings suggest that targeting autophagy may be a productive way of limiting EndMT.

### Therapeutic modulation of EndMT

**Fig. 1:**
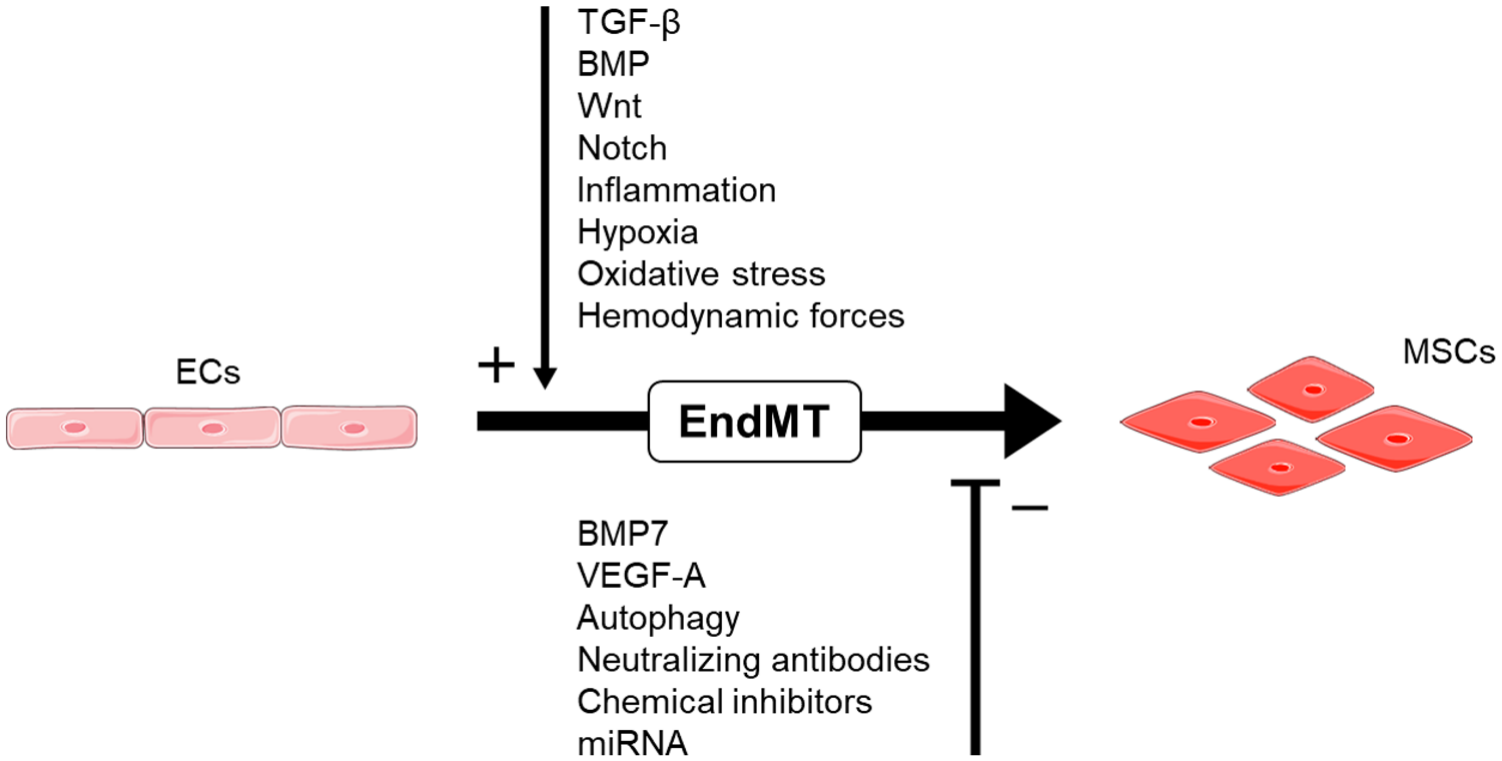
EndMT as a target for therapeutic intervention. ECs differentiate into MSCs via the process of EndMT, which is regulated by various signaling mechanisms. Numerous compounds can be used to block this differentiation step, thereby disrupting the process and potentially ameliorating the effects of pathological EndMT

Figure 1 highlights those signaling networks that could be plausible targets for therapeutically inhibiting EndMT as a treatment for several different pathologies. Neutralizing antibodies or chemical inhibitors targeting molecules required for EndMT could be an effective means of impeding the process [5]. Proof-of-principle evidence of this comes from experiments showing that inhibition of ALK5, TGF-βRII, β-glycan, and endoglin prevent embryonic EndMT in the endothelium of mice [96–98]. Consistently, reducing expression of EndMT regulators e.g. ALK2, ALK5, or Snail expression, resulted in a comparable block in EndMT in EC cultures [19, 24]. Another study has found that local and circulating ECs are capable of undergoing EndMT in response to musculoskeletal injury, suggesting that targeting early EC recruitment and trafficking could potentially impede pathological EndMT [99].

To date, several compounds have been tested, with mixed degrees of success, for their ability to inhibit EndMT. Many of these compounds interfere with TGF-β signaling. The dipeptidyl peptidase-4 (DPP-4) inhibitor, linagliptin, could block TGF-β2-induced EndMT by impairing its interaction with integrin β1 [100]. Arginylglycylaspartic acid (RGD) is an Arg-Gly-Asp tripeptide motif that is found in many matrix proteins and is responsible for integrin-dependent cell adhesion to the ECM. One recently developed RGD antagonist, RGD-2, was found to revert TGF-β1-induced EndMT and consequently has the potential to be employed as an anti-fibrotic therapeutic treatment [101]. A specific inhibitor of Smad3 (SIS3) was shown to block EndMT and reduce renal fibrosis [102]. EndMT was also inhibited by the ALK5 inhibitor SB-431542 in cultured ECs [9], and dorsomorphin blocked EndMT of endothelial cultures by inhibiting the kinase activity of a mutant ALK2 in FOP [19]. Celastrol was found to block TGF-β1-induced EndMT and has been promoted as a possible therapy for cardiac fibrosis [103]. TGF-β-induced EndMT was inhibited by kallistatin via upregulation of endothelial nitric oxide synthase (eNOS) and downregulation of EndMT-promoting miR-21 [104]. EndMT was also impaired by the angiotensin II type 1 receptor inhibitor losartan, which blocked TGF-β signaling [105]. Other compounds can disrupt EndMT by inhibiting different signaling pathways and/or intermediates (Table 1).

**Table 1:** Compounds modulating EndMT

Compound	Description	Mediator and/or signaling pathway^a^	Disease model^b^
Spironolactone	Aldosterone receptor inhibitor	Notch pathway	Fibrosis in human umbilical vein endothelial cells (HUVECs) [106]
Scutellarin	Flavone; major active component of breviscapine (natural plant extract)	Notch pathway	Isoprenaline (iso)-induced myocardial fibrosis in Sprague Dawley (SD) rats [107]
Bosentan, macitentan	ET-1 dual receptor antagonists	ET-1; TGF-β pathway	Murine lung microvascular endothelial cells (MVECs) and TGF-β1-induced tissue fibrosis in FVB/N mice [44]; systemic sclerosis (SSc)-derived ECs [45] SSc-derived fibroblast and MVEC co-cultures [108]
Rapamycin (sirolimus)	Immunosuppressive macrolide	mTOR pathway; possibly VEGF and MMPs	EA.hy926 cells [109]
Relaxin (RLX)	Protein hormone; regarded as anti-fibrotic	Notch pathway	Iso-induced cardiac fibrosis in SD rats [110]
Sulindac metabolites (sulindac sulfide and sulindac sulfone)	Non-steroidal anti-inflammatory drug (NSAID)	Wnt/β-catenin pathway; TGF-β pathway	Cerebral cavernous malformation (CCM) in endothelial *CCM3*-deficient mice [111]
Marimastat	MMP inhibitor	Wnt/β-catenin pathway	*Ex vivo* bovine corneal ECs [50]
Cinacalcet (CINA)	Calcimimetic agent	Serum parathyroid hormone (PTH)	Aortic calcification in uremic rats [112]
TAT-Y127WT	Mimic peptide	Protein phosphatase 2A (PP2A)	Nephropathy in mice; HUVECs [113]
Imatinib	PDGF receptor antagonist		PAH in rats [54]
Hydrocortisone	Hormone cortisol	Glucocorticoid receptor	Conditionally immortalized human brain microvascular endothelial cells (HBMEC/ciβ) [114]
Geniposide	Iridoid glycoside isolate from the gardenia plant	mTOR pathway	Bleomycin-induced SSc in HUVECs [115]

^a^The mediator(s) and/or signaling pathway(s) involved in the application of the listed compound

^b^The experimental in vitro or in vivo disease model used in the study

### EndMT in regenerative medicine and in vitro modeling applications

An extensive literature focused on the pathological consequences of EndMT should not overshadow several lines of evidence supporting the idea that EndMT could be harnessed for the purpose of tissue engineering, predicated on the fact that EndMT generates MSCs that can be programmed to differentiate into a wide variety of different cell types. In FOP, heterotopic bone is formed by a gain-of-function mutation in ALK2 [116]. Studies with lineage tracing and biomarker experiments have shown that this mutation causes ECs to undergo EndMT, thereby acquiring properties of MSCs [19, 117]. They have further demonstrated that these cells can be differentiated into osteoblasts, chondrocytes, or adipocytes [19]. The generation of osteoprogenitor cells via EndMT has also been seen in vascular [118], valvular [119], and tumor calcifications [120]. ECs were shown to be differentiated to chondrocytes via EndMT by high glucose levels [121], and ECs lining the vessels of white and brown adipose tissue have been shown to give rise to preadipocytes [122]. A number of studies have demonstrated the ability of ECs from vascular tumors to undergo EndMT in culture and form adipocytes, pericytes and smooth muscle cells (SMCs) [123], whilst related work has shown that EPCs can transform into smooth muscle cells [9]. The ability of ECs to form skeletal myocytes has also been observed during muscle repair [124]. ECs were also found to contribute to the cardiac renewal process [125].

**Fig. 2:**
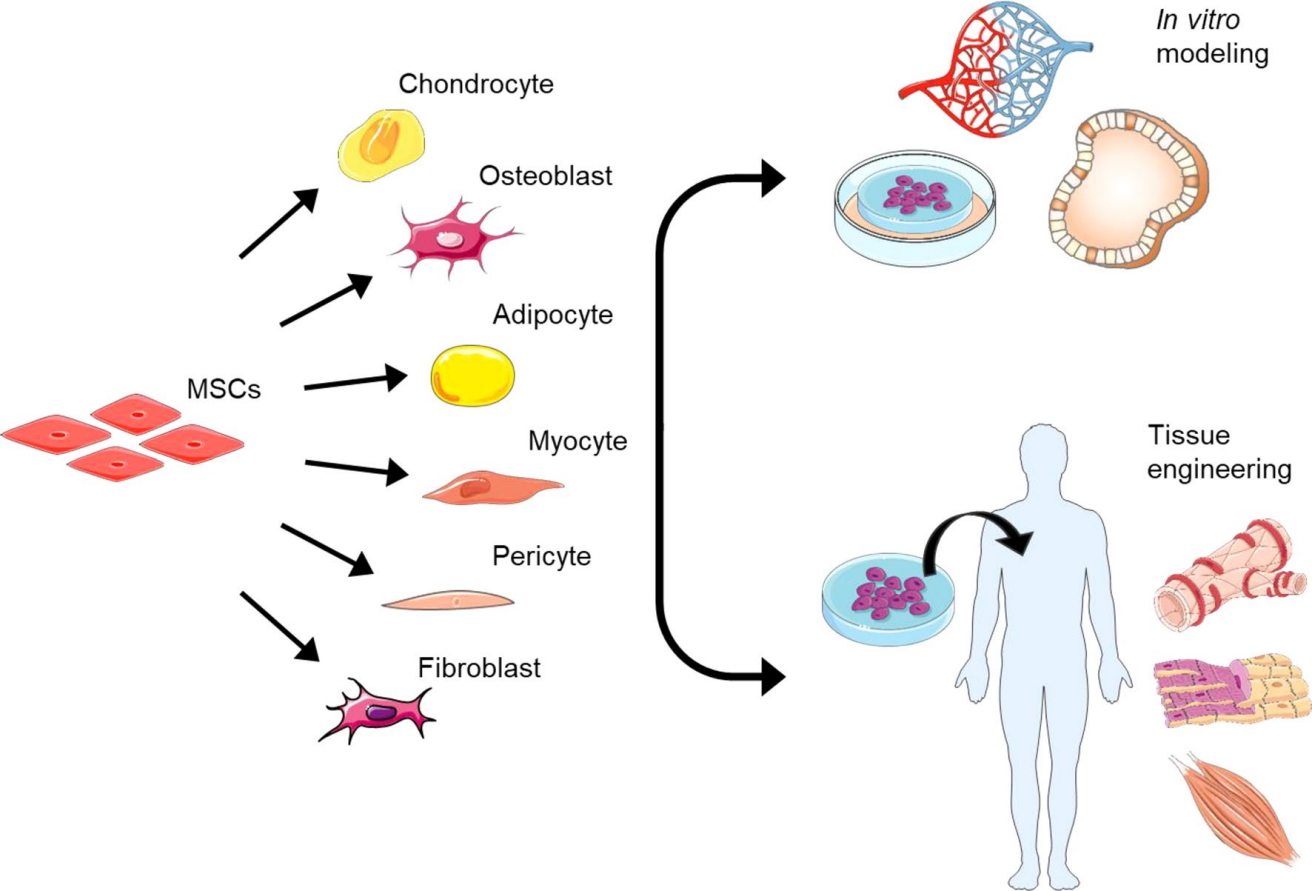
EndMT in tissue engineering and in vitro modeling. EndMT-derived MSCs can be differentiated into various mesenchymal cell types. Once the desired cell type is obtained, they can be used for tissue engineering and subsequent transplantation into the patient. The acquired cells can also be employed in experimental in vitro applications, such as in the construction of a vascularized 3D-organoid model

EndMT could thus be manipulated to generate multipotent MSCs from ECs, which can thereafter be transformed into different cell types. Via full or partial reprogramming, where intermediary cell types would suffice, EndMT could potentially be used in the treatment of a variety of diseases (Fig. 2). Bone disorders such as osteoporosis or osteoarthritis could be treated by EndMT-derived osteocytes or chondrocytes [20]. EndMT-mediated (cardio)myogenesis could be employed in the regeneration of cardiomyocytes after myocardial infarction [20]. Moreover, vascular tissue could be regenerated by EndMT via its ability to produce SMCs and pericytes [20]. Manipulating EndMT could offer a potential solution to controlling aberrant angiogenesis since expression of the EndMT-inducing transcription factor Slug was shown to regulate vessel sprouting [126]. Another study proposed that this angiogenic sprouting may represent a partial EndMT. Their results also clearly indicate the importance of the Snail family of transcription factors during angiogenesis [7], and suggest the involvement of EndMT, at least partially, in vasculature formation.

The appeal of employing EndMT in tissue engineering lies in the fact that the process can take place both *in vivo* and *ex vivo*. As pointed out by others, suitable drugs could be applied locally to degenerate tissues to reprogram ECs, which are present in abundance in any vascularized tissue, into the desired mesenchymal cell type [20]. To engineer tissues *ex vivo*, ECs can be isolated and, under the right 3D-culturing conditions, induced to undergo EndMT to become MSCs, using protocols more simple and cost-effective than those for induced pluripotent stem cells (iPSCs). After differentiating these stem cells into the cell type of interest, they can then be transplanted into the patient [20]. Perhaps not entirely unexpectedly, EndMT could also be beneficial in pathologies where fibrotic cells are actually desired, thus not requiring the additional step of differentiating MSCs to specialized cell types. One such study found that EndMT contributed to the therapeutic effects of bleomycin, a sclerosant used for the treatment of venous malformations (VMs), pointing to a possible role for this process in sclerotherapy [127]. Moreover, as ECM contributes to the mechanical functioning of cardiovascular tissue-engineered grafts, EndMT could aid the formation of cells, from ECs, that are capable of producing and remodeling ECM [128].

EndMT could also be employed for in vitro experimental purposes, such as the culturing and modeling of in vitro organs, which can be used as a substitute for experimental animal models (Fig. 2). One study established such an in vitro model with human embryonic stem cell (hESC)-derived ECs to study the regulation of Notch signaling in the induction of EndMT in cardiogenesis [129]. Another study generated an organoid-based EMT model from intestinal epithelial cells. These cells exhibited an in vivo physiology and, therefore, could be used to study EMT-associated intestinal fibrosis [130]. Such an approach could also be feasible for harvested ECs to study EndMT-related diseases. Although it is possible to grow organoids in vitro, a main restriction of 3D-culture systems is the lack of a vascular network [131].  In light of the fact that EndMT demonstrably plays a role in angiogenesis [126], cultured ECs could potentially be used to create vascular networks through EndMT, contributing to the development of a fully vascularized organoid.

### Concluding remarks

EndMT has an established role in many different pathologies. Targeting the signaling pathways responsible for EndMT could, therefore, be an effective means of facilitating wound healing as well as treating EndMT-associated diseases. This is, of course, not an easy undertaking, not least because EndMT is controlled by complex signaling networks and not simple, linear, and discrete signaling modules. This makes the selection of suitable therapeutic targets a far from trivial proposition. For instance, TGF-β could be an obvious candidate, however, it exerts pleiotropical effects in the regulation of a multitude of processes in various tissues and targeting this pathway could lead to major, unwanted side-effects [5]. Deciphering in greater depth, the activating and inhibitory EndMT signaling map could identify unique targets that offer realistic hopes of developing viable therapeutic strategies to modulate EndMT. Alongside a potential role in inhibiting pathological processes, EndMT could be exploited to play a more ‘creative’ role in tissue engineering. EndMT gives rise to multipotent MSCs that can be reprogrammed into various distinct cell types, offering the possibility that this capacity could be harnessed to advance regenerative medicine.
